# Proton and Heavy Particle Intracranial Radiosurgery

**DOI:** 10.3390/biomedicines9010031

**Published:** 2021-01-03

**Authors:** Eric J. Lehrer, Arpan V. Prabhu, Kunal K. Sindhu, Stanislav Lazarev, Henry Ruiz-Garcia, Jennifer L. Peterson, Chris Beltran, Keith Furutani, David Schlesinger, Jason P. Sheehan, Daniel M. Trifiletti

**Affiliations:** 1 Department of Radiation Oncology, Icahn School of Medicine at Mount Sinai, New York, NY 10029, USA; eric.lehrer@mountsinai.org (E.J.L.); kunal.sindhu@mountsinai.org (K.K.S.); stanislav.lazarev@mountsinai.org (S.L.); 2 Department of Radiation Oncology, UAMS Winthrop P. Rockefeller Cancer Institute University of Arkansas for Medical Sciences, Little Rock, AR 72205, USA; AVPrabhu@uams.edu; 3 Department of Radiation Oncology, Mayo Clinic, Jacksonville, FL 32224, USA; RuizGarcia.Henry@mayo.edu (H.R.-G.); Peterson.Jennifer2@mayo.edu (J.L.P.); Beltran.Chris@mayo.edu (C.B.); Furutani.Keith@mayo.edu (K.F.); 4 Department of Neurological Surgery, University of Virginia, Charlottesville, VA 22903, USA; DJS9C@hscmail.mcc.virginia.edu (D.S.); JPS2F@hscmail.mcc.virginia.edu (J.P.S.)

**Keywords:** radiosurgery, particle, carbon, proton, radiation therapy, radiation oncology, stereotactic, ablative, tumor, arteriovenous malformation

## Abstract

Stereotactic radiosurgery (SRS) involves the delivery of a highly conformal ablative dose of radiation to both benign and malignant targets. This has traditionally been accomplished in a single fraction; however, fractionated approaches involving five or fewer treatments have been delivered for larger lesions, as well as lesions in close proximity to radiosensitive structures. The clinical utilization of SRS has overwhelmingly involved photon-based sources via dedicated radiosurgery platforms (e.g., Gamma Knife^®^ and Cyberknife^®^) or specialized linear accelerators. While photon-based methods have been shown to be highly effective, advancements are sought for improved dose precision, treatment duration, and radiobiologic effect, among others, particularly in the setting of repeat irradiation. Particle-based techniques (e.g., protons and carbon ions) may improve many of these shortcomings. Specifically, the presence of a Bragg Peak with particle therapy at target depth allows for marked minimization of distal dose delivery, thus mitigating the risk of toxicity to organs at risk. Carbon ions also exhibit a higher linear energy transfer than photons and protons, allowing for greater relative biological effectiveness. While the data are limited, utilization of proton radiosurgery in the setting of brain metastases has been shown to demonstrate 1-year local control rates >90%, which are comparable to that of photon-based radiosurgery. Prospective studies are needed to further validate the safety and efficacy of this treatment modality. We aim to provide a comprehensive overview of clinical evidence in the use of particle therapy-based radiosurgery.

## 1. Stereotactic Radiosurgery

Stereotactic radiosurgery (SRS) blends radiation and neurosurgical techniques that were first proposed in 1951 at the Karolinska Institute in Stockholm, Sweden by Lars Leksell [1]. Stereotactic radiosurgery permits the delivery of an ablative dose of radiation in a highly conformal manner, allowing for minimization of dose to nearby tissues at risk. The original Gamma Knife^®^ radiosurgery platform (Elekta AB, Stockholm), which utilized cobalt-60 sources, was developed by Leksell and colleagues in the 1960s. Traditionally, SRS delivery required placement of a stereotactic frame; however, Cyberknife^®^ (Accuray, Sunnyvale), linear accelerator-based, and Gamma Knife ICON^®^ technologies have incorporated frameless stereotactic delivery.

SRS was historically defined as treatment delivery in a single session. However, fractionated stereotactic radiation therapy (FSRT) and staged SRS are becoming increasingly common, particularly for larger targets and those located in eloquent locations [2,3,4,5,6,7,8,9,10]. Stereotactic radiosurgery is commonly utilized for multiple intracranial pathologies, such as brain metastases [2,3,8,9,10,11,12,13,14,15,16], meningiomas [17,18,19,20,21,22,23,24], pituitary adenomas [25,26,27,28,29,30], trigeminal neuralgia [31,32,33,34], vestibular schwannomas [35,36,37,38,39,40,41,42,43,44,45,46,47], and arteriovenous malformations [48,49,50,51,52]. Side effects of SRS are varied but can include alopecia, fatigue, cerebral edema, and radiation necrosis [53]. Cognitive sequelae from SRS is a rare occurrence and is much more commonly associated with the use of whole brain radiation therapy (WBRT) [11,12,13,54,55,56,57].

In recent years, the use of SRS in the management of brain metastases and its role in immune system modulation, particularly when combined with immune checkpoint inhibitors, has generated a great deal of interest within the scientific community [2,8]. While the central nervous system (CNS) was long viewed as “immunologically privileged,” this view has recently been challenged by evidence suggesting that robust peripheral immune responses can result in penetration of peripheral antigens through the blood–brain barrier. Furthermore, there is evidence suggesting that neuroinflammation can further amplify these effects. Additionally, there has been evidence to suggest that ablative doses of radiation therapy, such as those used with SRS allow for enhanced CD8^+^ T-cell activation [58,59]. While a detailed analysis of this topic is out of scope for this manuscript, combining these two therapeutic modalities remains a pivotal area of research in CNS oncology.

In the forthcoming sections, we provide a comprehensive review of the physics of heavy particle radiation therapy and the role of heavy particle radiosurgery in the management of common pathologies of the CNS. Likely due to the increasing density of proton centers, the existing body of literature largely involves the use of proton radiosurgery [60,61,62,63,64,65,66,67,68].

## 2. The Proposed Benefits of Proton and Carbon Ion Radiosurgery

Fundamentally, the goal of radiation therapy is to administer a lethal dose to a tumor while sparing surrounding healthy tissues. X-rays, utilized in photon radiation therapy, impart energy in a relatively diffuse manner. Energy is deposited along the entire path of a photon beam as it traverses the body, and the maximum dose delivered is generally located just below the surface of the skin. In order to properly target tumors, treatment planners often employ multiple X-ray beams, the intensities of which are frequently modulated. While treatment planners attempt to spare normal tissues as much as possible in order to minimize the risk of side effects, structures surrounding treatment targets may still receive significant doses of radiation [69,70]. Thus, for tumors adjacent to critical structures, such as the optic chiasm or brainstem, photon-based radiosurgery may carry a significant risk of injury.

**Figure 1:**
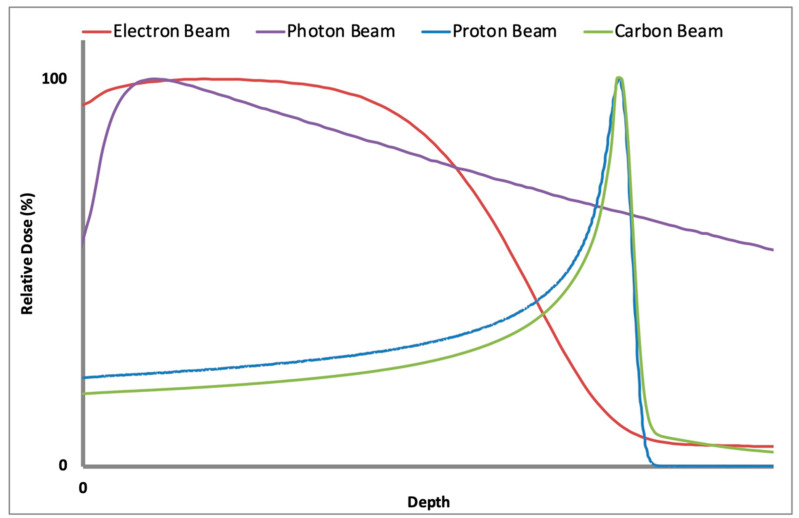
A graphical representation of tissue depth as a function of relative dose with the former on the *x*-axis and the latter on the *y*-axis. The purple graph depicts a photon beam, the red—an electron beam, the green—a carbon ion beam, and the blue—a proton beam. Both the proton and carbon ion beams exhibit significant Bragg Peaks, which are absent for photons and electrons. Additionally, the fragmentation tail associated with carbon ions is depicted. Note the absence of a sharp Bragg Peak for photons and its significant tissue penetration. Electron beams are largely used for superficial treatments (e.g., basal cell carcinomas of the skin) and exhibit a rapid dose fall-off and low tissue penetration.

Proton and carbon ion radiation therapy, by contrast, offer dosimetric advantages over photon radiation therapy that create additional therapeutic possibilities [71]. Unlike photons, protons and carbon ions generate an initial low dose region as they traverse through the body. At the ends of their paths, they deposit their maximum dose in a narrow region known as the Bragg Peak. Beyond the Bragg Peak, the dose delivered falls off dramatically. Carbon ion beams have narrower Bragg Peaks than proton beams, and each carbon ion beam generates a fragmentation tail—a low-dose region past the Bragg Peak that results from the fragmentation of carbon ions as they interact with matter. These fragmentation products continue on their path and deposit dose distal to the Bragg Peak. When utilizing carbon ion radiation therapy, planners must take fragmentation tails into account when generating treatment plans to avoid excess dose deposition in critical structures [70,72]. Figure 1 depicts the different depth dose profiles for photons, electrons, carbon ions and protons. Note the presence of a Bragg Peak for both carbon ions and protons, as well as the fragmentation tail associated with the former.

Carbon ion beams possess an additional advantage. They have narrower penumbras than proton beams, a difference that becomes more pronounced with depth. This results in sharper dose drop-offs at the lateral edges of carbon ion beams [72].

Collectively, the advantages of proton and carbon ion dosimetry potentially allow these radiation therapy techniques to better target tumors and spare surrounding normal structures than photon radiation therapy. In particular, the compactness of the dose delivery relative to photon radiotherapy may allow for the delivery of higher doses of radiation to tumors that are close to critical structures than would otherwise be possible [73] and may also offer relatively low risks of secondary malignancies [74].

Radiobiology may also favor heavy ion therapy; carbon ions are adroit at killing tumor cells. They demonstrate higher linear energy transfer than either photons or protons. As a result, they create complex, clustered DNA damage along their tracks, which is less easily repaired by tumor cells. In fact, the relative biological effectiveness (RBE) of carbon ions is approximately three times that of photons and protons [75,76]. Even hypoxic tumor cells, which show resistance to both photon and proton radiation therapy, show significant sensitivity to carbon ion radiation therapy [69]. The efficacy of carbon ion radiation therapy is also less dependent upon variations in the radiosensitivity of various phases of the cell cycle or diverse tissue types [76]. While distal doses are minimized with both protons and carbon ions, the greater RBE of carbon ions is demonstrated by the greater concentration of high dose within the gross tumor volume.

While most dosimetric characteristics favor particle radiotherapy over photon radiotherapy, there are a few potential drawbacks for particle radiotherapy. One major drawback is range uncertainty, which refers to uncertainty in the depth of the Bragg Peak due to tissue inhomogeneities, anatomical motion, and calculation uncertainties. Due to particle radiotherapy techniques dose being concentrated on the targeted tissue via the Bragg Peak, any uncertainty in depth requires compensation to prevent underdosing the targeted tissue and overdosing tissue beyond the expected region of the Bragg Peak. This uncertainty is often mitigated through the use of treatment margins around targeted tissue and nearby organs at risk [77]. For similar reasons, an exquisite attention to patient setup and organ motion is critical as misalignment of areas of low or high density can have significant impact on the resultant dose deposition. As radiosurgery often involves treating small targets, the dosimetry of small fields becomes an important source of treatment uncertainty for both photon and particle techniques. This is particularly important for particle radiosurgery (especially proton radiosurgery) due to the effect of multiple Coulomb scattering, which causes a slow but steady blurring of the aperture edge with depth due to small changes in the trajectories of individual particles [78]. However, this is only an issue for double scatter systems as most modern systems utilize spot scanning technology which does not have this limitation.

Passive scattering (including single and double scattering) requires the production of a spread-out Bragg Peak (SOBP), which permits dose delivery to the distal margins of the treatment target. This is accomplished by passing the particle beam through a filter. With the use of lead scattering foils, the particle beam develops a uniform energy profile. The beam then passes through a patient-specific aperture and compensator, which is designed so the distal range of the proton beam does not extend beyond the distal margin of the treatment target [72]. Active scanning (also known as pencil beam scanning) is a newer technology and utilizes orthogonal magnets, which allows for dose delivery as a series of small spots that each have their own Bragg Peak of varying energy and intensity [72,79,80]. This method allows for both distal and proximal dose conformality, the latter of which is sacrificed when utilizing passive scattering. Therefore, intensity modulated proton therapy (IMPT) is possible with active scanning but not with passive scattering [72,80].

An important component of radiosurgery is rigid immobilization that is reproducible. Some of the earliest applications of particle radiosurgery was with protons and deuterons and the Lawrence Berkeley National Laboratory for the treatment of pituitary tumors [81,82,83]. These early applications utilized a setup where the head was immobilized at the level of the sella with the use of a mask that was then tightened until the desired level of immobilization was reached. Similar immobilization systems were utilized in single or two-fraction helium ion radiosurgery of AVMs (arteriovenous malformations) [84,85]. However, any immobilization system, but particularly frame-based systems, should be considered carefully as they may limit beam angles in a way that has a much larger effect than in photon-based SRS. Subsequent developments in particle radiosurgery immobilization systems involved the incorporation of vacuum-enforced dental molds which would attach to the stereotactic frame. The Stereotactic Alignment in Radiosurgery (STAR) device at Massachusetts General Hospital allows for the head frame to be mounted to a rotating couch, allowing for increased degrees of freedom during treatment delivery [86].

While carbon radiosurgery has clear significant dosimetric advantages, its clinical application needs to be better elucidated. However, there are multiple retrospective series reporting on outcomes with proton radiosurgery, which are reviewed in the forthcoming sections [60,61,62,63,64,65,66,67,68].

## 3. The Drawbacks of Proton and Carbon Ion Radiosurgery

**Figure 2:**
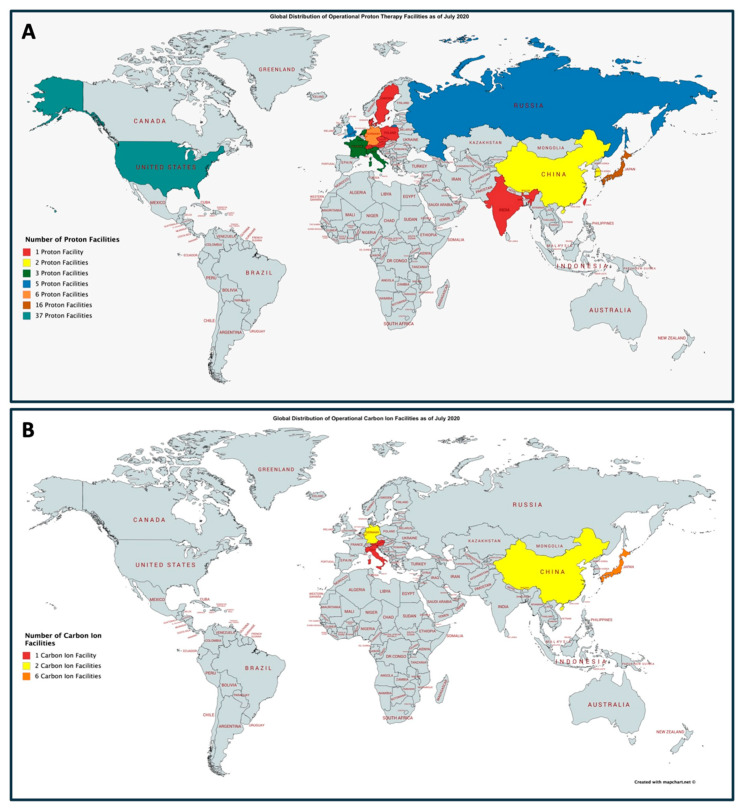
Global distribution of operation proton centers (**A**) and carbon ion centers (**B**) as of July 2020. Presently, there are 90 and 12 operational proton and carbon ion centers, respectively. Adopted from the Particle Therapy Co-Operative Group (https://www.ptcog.ch/index.php/facilities-in-operation). Figures generated using mapchart.net.

While there are potential dosimetric and radiobiological advantages in the utilization of protons and carbon ions in intracranial radiosurgery, there are several significant practical limitations in deploying these technologies. As shown in Figure 2A, there are a limited number of proton facilities throughout the world. As of July 2020, there are 90 operational proton therapy facilities throughout the world. The largest number of proton facilities are located in the United States with 37 presently operating. There is also marked limited availability of carbon ion therapy. Presently, there are 12 facilities that are operational throughout the world, as shown in Figure 2B. The largest concentration of carbon ion facilities is located within Japan with a total of six. While there are no carbon ion facilities presently operating in the United States, the Mayo Clinic in Jacksonville, Florida, has announced plans to construct a facility in the coming years.

Another major drawback to the use of proton and carbon ion therapy are costs associated with treatment. The cost of building a proton facility can cost over $200 million, while photon-based linear accelerators capable of utilizing intensity modulated radiation therapy and SRS often cost less than $10 million [87]. However, in recent years, the development of compact pencil beam scanning proton systems, such as the Mevion S250^®^, which commonly cost $20–30 million, have allowed for the implantation of this technology at a markedly lower cost, although still much higher than a photon-based SRS system.

While the cost of delivering proton therapy is often upwards of 50% greater than that of photon therapy, comparative effectiveness research studies have suggested that the lifetime cost of protons may be lower than that of photons when accounting for management of radiation toxicities, which is particularly pronounced in pediatric patients [88]. However, due to limited data comparing protons to photons and greater upfront costs, insurance approval for proton therapy remains a challenge for certain indications.

## 4. Brain Metastases

### 4.1. Background

The incidence of brain metastases is on the rise, which is largely due to improved systemic therapies and widespread adoption of magnetic resonance imaging (MRI) [89,90]. Approximately 200,000 patients are diagnosed with brain metastases annually in the United States and it is estimated that 10–30% of cancer patients go on to develop brain metastases [91,92,93]. Traditionally, treatment for brain metastases involved the use of corticosteroids and WBRT [94,95], however, the benefits of WBRT in the context of supportive care has come under more scrutiny in recent years [96]. Stereotactic radiosurgery has emerged as a highly effective treatment option in many patients with brain metastases and is associated with fewer instances of treatment-related cognitive deterioration [11,12,13].

### 4.2. Photon Radiosurgery

In 2009, Chang et al. published a phase III randomized study of 58 patients with 1–3 brain metastases who were randomized to SRS alone (*n* = 30) and SRS + WBRT (*n* = 28) [13]. This study closed early due to a high risk of decline in learning and memory in the combination therapy group (mean posterior probability of decline 52%) versus the SRS alone group (mean posterior probability of decline 24%). However, local and distant CNS progression favored the SRS + WBRT arm over the SRS arm, with 73% and 27% of patients being free from CNS progression in the SRS + WBRT and SRS arms, respectively (*p* = 0.0003). In 2017, Brown et al. published the results of the N107C, a phase III randomized trial, which randomized patients with a single resected brain metastasis to adjuvant SRS or WBRT [11]. This study found a longer cognitive-deterioration-free survival favoring the SRS arm (HR: 0.47; 95% CI: 0.35–0.64; *p* < 0.0001). Additionally, cognitive deterioration at 6 months was 52% versus 85% (*p* < 0.0003) in the SRS versus WBRT arms, respectively. However, 12-month local control 61.8% versus 87.1% (*p* = 0.00068) and 12-month distant brain control 64.7% versus 89.2% (*p* = 0.00045) both favored the WBRT arm. Therefore, while SRS is associated with less cognitive sequelae and improved quality of life compared to WBRT, the likely need for retreatment is high, necessitating the need for regular follow-up volumetric MRI scans.

### 4.3. Particle Radiosurgery

Despite the widespread use of photon-based radiosurgery, there is a paucity of data available regarding the use of particle radiosurgery in the management of brain metastases. In 2018, the Massachusetts General Hospital published a retrospective series of 370 patients with 815 brain metastases that were treated with proton radiosurgery at the Harvard Cyclotron Laboratory (Cambridge, MA, USA) [60]. The median prescription dose was 17.3 GyRBE which was prescribed to the 90% isodose line. Strict brainstem and optic chiasm dose constraints were used. The median prescription target volume was 0.6 cc. With a median follow-up of 9.2 months, local and distant brain failure at 12 months was 8.5% (95% CI: 6.7–10.6) and 48.2% (95% CI: 43–53.2%). No grade 4 or 5 acute toxicities occurred, 40.5% (150 patients) experienced acute toxicities, the majority being grade 1 (109 patients), grade 2 (22 patients), and grade 3 (19 patients). Sixty-five patients underwent a craniotomy after proton SRS, 61.5% of whom were symptomatic prior to surgery and 29.2% of whom experienced asymptomatic radiographic progression. Pathologic radiation necrosis was found in 40% (*n* = 26) of these patients, of whom 54.8% (*n* = 14) were symptomatic prior to surgery. The 12-month cumulative incidence of radiation necrosis was 3.6% (95% CI: 2–5.8%). Patients who went on to develop radiation necrosis had a median SRS target volume of 2.1 cc. The only factor significantly associated with radiation necrosis on multivariate analysis was target volume size (HR: 1.13; 95% CI: 1.07–1.20). Further details are presented in Table 1 and Table 2.

## 5. Pituitary Adenoma

### 5.1. Background

Pituitary adenomas are a relatively common benign CNS neoplasm and account for roughly 10–20% of newly diagnosed brain tumors [99]. Approximately 12,000 cases are diagnosed annually in the United States with an incidence of 3–4 per 100,000 [100]. Pituitary adenomas are classified by their endocrine status, either as functional or nonfunctional, with the former referring to hormone hypersecretion and the latter referring to an absence of hormone production. Approximately 75% of pituitary adenomas are functional, the most common being prolactinomas [101]. While recurrence rates are highly dependent upon the extent of surgical resection, size of the tumor, and endocrine status, recurrence rates ranging from 10–60% have been observed [102,103,104,105]. Therefore, radiation therapy is a commonly used modality in the salvage setting. The choice of conventionally fractioned RT versus SRS is typically dictated by distance of the tumor from the anterior optic pathways. The former is commonly preferred for distances between the tumor and the optic chiasm of <3–5 mm, although consideration of hormonal status and visual symptoms is critical.

### 5.2. Photon Radiosurgery

Contemporary studies involving the use of photon SRS in the management of functional and non-functional pituitary adenomas have demonstrated excellent rates of local control with minimal rates of visual toxicity. In 2013, Sheehan et al. published a multicenter study of 512 patients diagnosed with nonfunctional pituitary adenomas who were treated with SRS to a mean dose of 16 Gy [30]. With a median follow-up of 36 months, 5-year tumor control rates were excellent at 95% with a 6.6% rate of visual toxicity and a 21% rate of hypopituitarism. In 2013, Sheehan et al. published their findings from the University of Virginia Gamma Knife Center, where 96 patients with Cushing’s Disease were treated with SRS to a mean dose of 16 Gy [29]. The tumor control rate at 5-years was 98% with hormone control rates of 70%. Visual toxicity was observed in 5.2% and hypopituitarism in 36%.

### 5.3. Particle Radiosurgery

The particle radiosurgery experience in the management of pituitary adenomas is largely limited to the use of protons for functional lesions [62,63]. In 2007, Petit et al. published a retrospective series of 22 patients treated with proton SRS between 1992–2003 for persistent acromegaly at the Harvard Cyclotron Laboratory (Cambridge, MA, USA) [62]. All patients underwent transsphenoidal resection prior to SRS and median follow-up was 6.3 years (range: 2.5–14.2 years). The median dose was 20 CGE (range: 15–24 CGE). A complete response, which was defined as a sustained (≥3 month) normalization of insulin-like growth factor-1 without medical suppression, was observed in 59% of patients. No new visual complications, seizure disorders, brain injuries or secondary brain tumors were noted on follow-up imaging. Eight patients (38%) developed new hypopituitarism. In 2008, the same group published a retrospective study of 38 patients with Cushing’s Disease (*n* = 33) or Nelson’s Syndrome (*n* = 5) who previously underwent transsphenoidal surgery without biochemical cure and received proton SRS to a dose of 20 CGE (range: 15–20 CGE) [63]. In the Cushing’s Disease cohort, 52% achieved a complete response at a median time of 14 months post-SRS, 36% achieved stable urine cortisol levels but required continued medical therapy without tumor regrowth, and 12% had persistently elevated urine cortisol levels and radiographic evidence of disease progression that necessitated additional surgery. In the Nelson’s syndrome cohort, 100% achieved a complete response to treatment. No new visual complications were observed in any patients and 52% required hormone supplementation for hypopituitarism. Further details are presented in Table 1 and Table 2.

## 6. Vestibular Schwannoma

### 6.1. Background

Vestibular schwannomas (also commonly referred to as an acoustic neuroma) are benign Schwann cell-derived tumors involving the vestibular division of the vestibulocochlear nerve. The incidence of vestibular schwannomas in the general population is roughly 1:100,000 [106,107]. The most well-documented risk factor for the development of vestibular schwannomas is in patients diagnosed with neurofibromatosis type 2 (NF2); however, these patients typically develop bilateral disease as well as multiple other tumors [108]. Patients commonly present with tinnitus, gait abnormalities, vertigo, and hearing loss. Pain is a less common clinical manifestation and is due to involvement of the trigeminal nerve. Treatment approaches for vestibular schwannoma include SRS, conventional RT, surgery, and observation.

### 6.2. Photon Radiosurgery

Stereotactic radiosurgery is associated with excellent rates of tumor control. In 2013, Hasegawa et al. published a retrospective study of 440 patients with vestibular schwannoma who were treated with Gamma Knife radiosurgery [42]. With a median follow-up of 12.5 years, the 5- and 10-year progression-free survival was 93% and 92%, respectively. No patients experienced a treatment failure more than 10 years after treatment. The 10-year facial nerve preservation rate was 97% in patients treated to a margin dose >13 Gy and 100% in patients treated to a margin dose of ≤ 13Gy. Ten patients (2.3%) developed delayed cyst formation.

### 6.3. Particle Radiosurgery

In 2003, Weber et al. published a retrospective series of 88 patients with vestibular schwannoma who were treated with proton SRS at the Harvard Cyclotron Laboratory (Cambridge, MA) [68]. The median dose was 12 CGE (range: 10–18 CGE) delivered over a median of 3 fractions (range: 2–4). The median tumor volume was 1.4 cc. Twenty-one (24%) and 67 (76%) patients had Gartner–Robertson Grade 1 or 2 versus Grade 3–5 or unknown hearing loss, respectively. Seventy-nine (89.8%) and nine (10.2%) patients were House–Brackmann Grade 1 or 2–5, respectively, and nine (10.2%) patients presented with trigeminal nerve dysfunction. Five-year tumor control rate was 93.6% (95% CI: 88.3–99.3%). Seven of 21 patients retained functional hearing after treatment, and 5-year facial nerve function preservation was 91.1% (95% CI: 85–97.6%). The 5-year trigeminal nerve preservation rate was 89.4% (95% CI: 82–96.7%). One patient on anticoagulation developed an extratumoral hemorrhage within the choroid plexus that required a craniotomy 19.1 months after proton SRS. Further details are presented in Table 1 and Table 2.

## 7. Meningioma

### 7.1. Background

Meningiomas are the most commonly diagnosed brain tumors in adults, representing approximately 20–30% of primary brain tumor diagnoses, and are seen in 7.8 per 100,000 people [100,109,110]. They arise from arachnoid cap cells within the dura and are largely benign (WHO Grade I) in nature. However, atypical (WHO Grade II) and anaplastic (WHO Grade III) meningiomas are associated with more aggressive behavior. Surgical intervention aimed at achieving a gross total resection is the standard treatment; however, the risk of recurrence remains high. Radiation therapy is commonly employed in both the definitive, adjuvant, and salvage settings.

### 7.2. Photon Radiosurgery

The role of SRS is largely in the definitive, adjuvant, and salvage settings for WHO Grade I meningiomas, while conventionally fractionated radiation therapy is generally preferred for WHO Grade II and Grade III lesions. In 2008, Kondziolka et al. published a study conducted at The University of Pittsburgh, where 972 patients with 1045 intracranial meningiomas were treated with SRS to a mean margin dose of 14 Gy over an 18-year period [23]. The overall tumor control rate in patients with WHO Grade I meningiomas was 93%, while control rates of 50% and 17% were observed in patients with WHO Grade II and Grade III meningiomas, respectively. Complications attributable to radiosurgery were observed in 76 patients (7.7%) at an average time until presentation of 11 months. In 2002, a multicenter study published by Santacroce et al. evaluated 4565 patients with 5300 benign meningiomas across 15 institutions who were treated with SRS to a median margin dose of 14 Gy [24]. With a median imaging follow-up of 63 months, an overall control rate of 92.5% was observed. Additionally, permanent morbidity was seen in 6.6% of patients following SRS.

### 7.3. Particle Radiosurgery

In 2011, Halasz et al. published a prospective study where 51 patients with 50 benign meningiomas were treated with proton radiosurgery at the Harvard Cyclotron Laboratory (Cambridge, MA, USA) [61]. Patients were treated to a median dose of 13 GyRBE (range: 10–15.5 GyRBE) prescribed to the 90% isodose line with a median prescription volume of 4.4 cc (range: 1.1–33 cc). With a median follow-up of 32 months, a 3-year tumor control rate of 94% (95% CI: 77–98%) and a median time to progression of 48 months was observed. Three patients (5.9%) experienced transient adverse effects, including facial pain and seizures that resolved with steroids and antiepileptic medications, respectively. Three patients (5.9%) developed permanent adverse effects, which included seizures in two patients requiring long-term use of antiepileptic medications and panhypopituitarism in another. No new cranial nerve deficits were observed after proton SRS. On univariate analysis, the presence of atypical histologic features was associated with a greater risk of tumor recurrence (*p* = 0.03). Further details are presented in Table 1 and Table 2.

## 8. Arteriovenous Malformation

### 8.1. Background

Arteriovenous malformations are vascular lesions that occur when high pressure arterial blood is shunted to the low-pressure venous system without passing through a capillary network. These malformations have a risk of spontaneous rupture over time. Autopsy studies have demonstrated a 0.6% prevalence of intracranial AVMs [111]. The most widely-used system for grading AVMs is the Spetzler–Martin scale, which is graded from 1–5 with higher grades correlating with worse outcomes [112]. Stereotactic radiosurgery is a treatment option for AVMs, which has been shown to successfully achieve lesion obliteration in many clinical scenarios. However, a major limitation of SRS in this setting is that AVM obliteration can take up to 2–4 years following treatment [113].

### 8.2. Photon Radiosurgery

In 2014, Kano et al. published a study of 474 patients with Spetzler–Martin Grade 3 AVMs who were treated with SRS at the University of Pittsburgh [114]. This study further stratified patients into Grade 3a (59%), 3b (9%), and 3c (31%) subgroups. With a median follow-up of 89 months, total obliteration rates based on angiographic or MRI criteria were 48%, 69%, and 72% at 3-, 4-, and 5-years post-SRS, respectively. The cumulative hemorrhage rate at 10-years was 9%. In 2005, Maruyama et al. published a retrospective study of 500 patients with cerebral AVMs who were treated with Gamma Knife radiosurgery with a median Spetzler–Martin Grade of 3 [115]. This study was designed to assess the rate of hemorrhage that occurred before radiosurgery, between radiosurgery and obliteration (latency period), and after obliteration. Forty-two hemorrhages occurred before radiosurgery, 23 during the latency period, and 6 following obliteration. The risk of hemorrhage decreased by 54% during the latency period compared to before radiosurgery (HR: 0.46; 95% CI: 0.25–0.80; *p* = 0.006) and by 88% after obliteration compared to before radiosurgery (HR: 0.12; 95% CI: 0.05–0.29; *p* < 0.001).

### 8.3. Particle Radiosurgery

In 1994, Seifert et al. published a retrospective series of 68 patients treated between 1980–1990 who were treated with proton SRS [64]. Their results were associated with a significant amount of morbidity and mortality, where 28.6% of patients experienced worsening of symptoms after SRS, such as increased frequency of seizures (7 patients), hemorrhage of the irradiated AVM (6 patients of whom two died), and progressive neurologic deficits (6 patients). Additionally, no AVM larger than 3 cm was successfully obliterated with SRS. In 2005, Vernimmen et al. published a retrospective series of 64 patients who were treated for cerebral AVMs with proton SRS from 1993–2003 [67]. The median AVM volume was 16.3 cc (range: 1.7–110.6 cc); 3%, 17%, 30%, 42%, and 8% of AVMs were Spetzler–Martin Grade 1, 2, 3, 4, and 5, respectively, and the median follow-up was 62 months. Patients were treated in 2–3 fractions. Dose was based on AVM size: mean dose 27.2 SFEcGyE (<10 cc), mean dose 23.2 SFEcGyE (10–13.9 cc), and mean dose 27 SFEcGyE (≥14 cc). Patients were stratified by AVM size into two groups: (1) <14 cc and (2) ≥14 cc. In the former, 67% and 17% achieved an obliteration and partial response, respectively. In the latter, 43% and 21% achieved an obliteration and partial response, respectively. Transient late effects were observed in 23% of patients and 6% developed a permanent late Grade 3 or 4 toxicity, such as epilepsy and permanent neurologic deficits. In 1993, Barker et al. published a retrospective series of 1250 patients treated with proton SRS between 1965–1993 at the Harvard Cyclotron Laboratory (Cambridge, MA, USA) [97]. Patients were treated with a single fraction median dose of 10.5 Gy (range: 4–65 Gy). Treatment-related complications consisted of hemiparesis in 24 patients, visual field deficits in 11 patients, cognitive dysfunction in 8 patients, ataxia in 4 patients, speech deficits in 3 patients, hemisensory deficits in 2 patients, and hearing loss in 1 patient. Additionally, multiple patients had more than one of the aforementioned complications, three had a global deterioration in neurologic function, and four had a disabling increase in headache severity. Complications presented 0.2–6.8 years after treatment with a 10-year actuarial incidence of 4.5%.

In 1990, Steinberg et al. published a retrospective study of 35 patients treated from 1983–1989 with helium ion radiosurgery to cerebral angiographically occult vascular malformations [98]. Initially, high doses of 45 GyE were used, which were subsequently changed to 10–15 GyE. While the data were limited, 27 patients in “excellent” or “good” condition prior to SRS remained stable or improved neurologically, 2 patients in “poor” condition who received previous fractionated RT died at 9- and 14-months post-SRS, 7 patients experienced neurologic deterioration due to recurrent AVM hemorrhage, and 4 patients worsened clinically 3–12 months after SRS. Further details are presented in Table 1 and Table 2.

## 9. Conclusions

Stereotactic radiosurgery with the use of photons is a highly effective and safe treatment modality for managing multiple benign and malignant intracranial pathologies. There exists limited data utilizing particle radiosurgery for intracranial lesions. The vast majority of available data are single institution retrospective studies that utilized protons. Furthermore, there is a marked paucity of data exploring the use of heavy particles in the radiosurgical setting. While there are theoretical benefits to using particles over photons for intracranial radiosurgical applications, prospective data are needed to validate the safety and efficacy of this treatment modality. Presently, there are a great paucity of prospective trials studying this therapeutic modality in the intracranial setting. However, there has been an increased focus on utilizing this therapy in extracranial locations. As such, there are presently clinical trials underway utilizing proton stereotactic body radiation therapy in the management of NSCLC and liver metastases. Particle-based radiosurgery offers opportunities to improve the therapeutic ratio through, amongst other factors, dose escalation to the target, reduction in dose to surrounding tissues, and more favorable radiobiological effects, regardless of target tissue type or cell cycle. However, the therapeutic ratio remains excellent for photon radiosurgery and further studies aimed at comparative effectiveness between these two modalities are needed to further elucidate the validity these hypotheses. In select patients who have received previous radiation therapy and have lesions located in highly eloquent locations, particle radiosurgery may be a suitable alternative to photons. Additionally, further study exploring dose relationships in the setting of proton SRS are needed.

## Figures and Tables

**Table 1:** Patient and treatment details in select studies utilizing particle radiosurgery.

Author, Year	Target	Particle	*n* (M/F)	Years	Patient Details	Treatment Details
Atkins et al., 2018 [60]	Brain Metastases	Protons	370 (170/200) (815 BrM)	1991–2016	Median age (range): 61 (20–93) Histology: NSCLC: 34.1% (*n* = 126); Melanoma: 28.1% (*n* = 104); Breast: 17.3% (*n* = 64); RCC: 4.9% (*n* = 18); HN: 3.8% (*n* = 14); SCLC: 3.5% (*n* = 13); GI: 3.5% (*n* = 13); GYN: 2.2% (*n* = 8); other: 2.7% (*n* = 10) Prior cranial RT: 54.9% (*n* = 203) Neurologic symptoms at presentation: 51.6% (*n* = 191) Baseline KPS: 50–70: 11.1% (*n* = 41); 80–100: 67.6% (*n* = 250) Median target volume: 0.6 cc (range: 0.02–23.3 cc) Median F/U: 9.2 mos Institutions: Harvard Cyclotron Laboratory (Cambridge, MA, USA) and Francis H. Burr Proton Therapy Center at Massachusetts General Hospital (Boston, MA, USA)	Median dose: 17.3 GyRBE Concurrent systemic therapy: 23.6% (*n* = 192) (chemotherapy: 39.6% (*n* = 76), immunotherapy: 17.2% (*n* = 33), targeted therapy: 33.9% (*n* = 65), unknown: 9.4% (*n* = 18))
Sikuade et al., 2015 [65]	Choroidal Melanoma	Protons	106 (64/43)	2001–2011	Median age (range): 59 (24–82) Median tumor diameter: 11 mm (range: 3.6–20.8 mm) Median tumor thickness: 4 mm (range: 1–11.6 mm) Median distance to the optic disc: 2 mm (range: 0–15 mm) Median F/U: 29 mos (range: 7–95 mos) Institution: Douglas Cycloton Clatterbridge Cancer Centre (Wirral, UK)	59.4 CGE in 4 daily fractions
Halasz et al., 2011 [61]	Benign Meningioma	Protons	50 (15/35) (51 tumors)	1996–2007	Median age (range): 60 (33–85) Location: posterior fossa: 24% (*n* = 12); middle fossa: 45% (*n* = 23); convexity: 6% (*n* = 3); other: 26% (*n* = 13) Presenting symptoms: none: 6% (*n* = 3); cranial neuropathy: 58% (*n* = 29); seizures: 12% (*n* = 6); dizziness: 10% (*n* = 5); headaches: 8% (*n* = 4); sensory deficit: 4% (*n* = 2); motor deficit: 4% (*n* = 2); other: 2% (*n* = 1); unknown: 6% (*n* = 3) Prior surgery: none: 64% (*n* = 32); STR: 28% (*n* = 14); GTR: 8% (*n* = 4) Prior RT: none: 96% (*n* = 48); fractionated: 4% (*n* = 2) Median F/U: 32 mos (range: 6–133 mos) Institutions: Harvard Cyclotron Laboratory (Cambridge, MA, USA) and Francis H. Burr Proton Therapy Center at Massachusetts General Hospital (Boston, MA, USA)	Median dose: 13 GyRBE (range: 10–15.5 GyRBE) prescribed to the 90% isodose line D_max_ brainstem ≤ 12 GyRBE D_max_ optic chiasm ≤ 8 GyRBE Median prescription volume: 4.4 cc (range: 1.1–33 cc)
Petit et al., 2008 [63]	Pituitary Adenoma (ACTH-producing)	Protons	38 (6/32)	1992–2005	Median age (range): 42 (14–60) Cushing disease (*n* = 33) and Nelson syndrome (*n* = 5), all patients underwent transsphenoidal surgery without biochemical cure, *n* = 4 had prior proton RT, and all Nelson syndrome patients underwent bilateral adrenalectomy 29–228 months prior to proton SRS Median F/U: 62 mos (range: 20–136 mos) Institution: Massachusetts General Hospital (Boston, MA, USA)	Median dose: 20 CGE (range: 15–20 CGE) Prescribed at the 90% isodose line in *n* = 37 and the 100% isodose line in *n* = 1 Delivered in a single fraction D_max_ to the optic chiasm < 8 CGE
Petit et al., 2007 [62]	Pituitary Adenoma (acromegaly)	Protons	22	1992–2003	Median age (range): 38 (18–53) All patients underwent prior transsphenoidal surgery without biochemical cure Median F/U: 6.3 years (range: 2.5–14.2 years) Institution: Massachusetts General Hospital (Boston, MA, USA)	Median dose: 20 CGE (range: 15–24 CGE)
Vernimmen et al., 2005 [67]	Cerebral AVM	Protons	64 (40/24)	1993–2003	Median age (range): 35 (7–59) <14 cc: 41% (*n* = 26); ≥14 cc: 59% (*n* = 38) AVM median volume: 16.3 cc (range: 1.7–110.6 cc) Presenting symptoms: headaches: 22% (*n* = 14), hemorrhage: 55% (*n* = 35), seizures: 30% (*n* = 19); neurological deficit: 6% (*n* = 4) SM Grade 1: 3% (*n* = 2), 2: 17% (*n* = 11), 3: 30% (*n* = 19), 4: 42% (*n* = 27), 5: 8% (*n* = 5) Median F/U: 62 mos Institution: iThemba Laboratory for Accelerator Based Sciences (LABS), Cape Town, South Africa	Mean dose (<10 cc): 27.2 SFEcGyE Mean dose (10–13.9 cc): 23.2 SFEcGyE Mean dose (≥14 cc) 27 SFEcGyE
Weber et al., 2003 [68]	Vestibular Schwannoma	Protons	88 (46/42)	1992–2000	Median age (range): 69 (36–92) Prior resection: 17% (*n* = 15); STR: 8% (*n* = 7), GTR: *n* = 9% (*n* = 8) Tumor volume: median 1.4 cc (range: 0.1–15.9 cc) GR Grade 1 or 2: 24% (*n* = 21); 3–5 or not tested 76% (*n* = 67) HB Grade 1: 89.8% (*n* = 79); 2–5: 10.2% (*n* = 9) Trigeminal nerve dysfunction: 10.2% (*n* = 9) Median F/U: 38.7 mos (range: 12–102.6) Institution: Harvard Cyclotron Laboratory (Cambridge, MA, USA)	Median dose: 12 CGE (range: 10–18 CGE) Median maximum tumor dose: 17.1 CGE (range: 13.3–20 CGE) Median number of fractions: 3 (range: 2–4) Number of isocenters: 1
Barker et al., 2003 [97]	Intracranial AVM	Protons	1250	1965–1993	Median age: 31 (0.02–69 years) Location: frontal lobe: 20% (248); parietal lobe: 36% (445); temporal lobe: 245 (20%); thalamus: 25% (316); brainstem: 4% (53); cerebellum: 6% (76) Median treatment volume: 33.7 cm^3^	Median dose: 10.5 Gy (range: 4–65 Gy) delivered in a single fraction
Silander et al., 2002 [66]	Cerebral AVM	Protons	26 (14/12)	1991–1997	Median age (range): 38 (23–64) Nidus volume: 0.3–102 cc (median: 13 cc) SM Grade: 1–2 (*n* = 10); 3–5 (*n* = 14) Median F/U: 40 mos (range: 33–62) Institution: The Svedberg Laboratory (Uppsala, Sweden)	20–25 Gy/2–4 fractions (nidus < 30 cc or > 30 cc) (*n* = 19) 21–25 Gy/4 fractions (nidus > 30 cc) (*n* = 6) 16 Gy/4 fractions (prior RT with helium ions) (*n* = 1)
Seifert et al., 1994 [64]	Cerebral AVM	Protons	68 (30/38)	1980–1990	Mean age (range): 33 (14–60) 26.9% (*n* = 17) <3 cm; 58.7% (*n* = 37); 3–6 cm; 14.2% (*n* = 9) >6 cm SM Grade I: 4.4% (*n* = 3); II: 5.9% (*n* = 4); III: 42.6% (*n* = 29); IV: 36.8% (*n* = 25); V: 2.9% (*n* = 2); VI: 7.4% (*n* = 5) Presenting symptoms: seizures (*n* = 39); hemorrhage (*n* = 32); progressive neurological deficits (*n* = 16); headache (*n* = 9); incidental finding (*n* = 3) F/U available for 92.6% (*n* = 63) Institutions: Various radiosurgical centers in the United States	NR
Steinberg et al., 1990 [98]	Cerebral AVM	Helium	35 (20/15)	1983–1989	Age range: 13–64 Location: brainstem: 54% (*n* = 19); thalamus/internal capsule: 26% (*n* = 9); basal ganglia: 9% (*n* = 3); motor/language area: 9% (*n* = 3); cerebellopontine angle: 2% (*n* = 1) Mean F/U: 40 mos (range: 1–7 years) Institution: University of California (Berkeley, CA, USA)	Initially, high doses of 45 GyE were used, which were changed to 10–15 GyE

Abbreviations: ACTH: adrenocorticotropin hormone; AVM: arteriovenous malformation; BrM: brain metastases; cc: cubic centimeter; CGE: cobalt gray equivalents; F: number of female patients; F/U: follow-up; GI: gastrointestinal; GR: Gardner–Robertson; GTR: gross total resection; Gy: gray; GYN: gynecologic; HB: House– Brackmann; HN: head and neck; KPS: Karnofsky performance status; M: number of male patients; mos: months; *n*: total number of patients; NR: not reported; NSCLC: non-small cell lung cancer; pts: patients; RBE: relative biological effectiveness; RT: radiation therapy; SCLC: small cell lung cancer; SFEcGyE: single-fraction equivalent cobalt gray equivalent dose; SM: Spetzler–Martin; STR: subtotal resection; USA: United States of America.

**Table 2:** Patient and treatment details in select studies utilizing particle radiosurgery.

Author, Year	Outcomes
Atkins et al., 2018 [60] (Brain Metastases)	Local Failure: 6-month: 4.3% (95% CI: 3–5.9%), 12-month: 8.5% (95% CI: 6.7–10.6%) Distant Brain Failure: 6-month: 39.1% (95% CI: 34.1–44%), 12-month: 48.2% (95% CI: 43–53.2%) Overall Survival: 6-month: 76% (95% CI: 71.3–80%), 12-month: 51.5% (95% CI: 46.3–56.5%) Median Survival: 12.4 months (95% CI: 10.8–14 mos) after proton SRS, 18.1 mos (95% CI: 16.1–20.1 mos) after diagnosis Acute toxicity (within 8 weeks of treatment): no grade 4 or 5 toxicities; 40.5% (*n* = 150) experienced grade 1–3 acute toxicities: grade 1: 72.9% (*n* = 109), grade 2: 14.7% (*n* = 22), grade 3: 12.7% (*n* = 19) Radiation necrosis ○ 65 patients underwent craniotomy after proton SRS, where 61.5% (*n* = 40) were symptomatic prior to surgery and 29.2% (*n* = 19) had asymptomatic radiographic progression; pathologic RN was found in 40% (*n* = 26), of whom 54.8% (*n* = 14) were symptomatic prior to surgery ○ Median time to pathologically confirmed RN from proton SRS: 12 mos (range: 1.1–8.2 years) ○ 12-month cumulative incidence: 3.6% (95% CI: 2–5.8%) ○ Median proton SRS target volume: 2.1 cc ○ On multivariate analysis, target volume (HR: 1.13; 95% CI: 1.07–1.20) was the only statistically significant covariate
Sikuade et al., 2015 [65] (Choroidal Melanoma)	Treatment complications ○ 5% of patients had significant visual loss during the F/U period ○ 30% (*n* = 31) developed radiation retinopathy ○ 13% (*n* = 14) developed optic neuropathy ○ 5% (*n* = 5) developed rubeotic glaucoma and 2 required enucleation 2.8% (*n* = 3) experienced a local recurrence 93.5% eye retention rate
Halasz et al., 2011 [61] (Benign Meningioma)	Tumor control ○ Stable size: 65% (*n* = 33), decreased size: 25% (*n* = 13), increased size: 10% (*n* = 5) ○ 3-year tumor control rate for benign meningiomas: 94% (95% CI: 77–98%) ○ Median time to progression: 48 mos (range: 23–109 mos) ▪ 4/5 pts had prior resection ▪ 1/5 pt had prior fractionated photon RT ▪ 2/5 pts had atypical features on pathology ▪ Atypical features were associated with tumor recurrence on univariate analysis (*p* = 0.03) Clinical outcomes assessed in 34 patients ○ 47% (*n* = 16) symptomatic improvement ○ 44% (*n* = 15) unchanged symptoms ○ 9% (*n* = 3) worsened symptoms ▪ 2/3 pts had tumor progression Morbidity ○ 5.9% (*n* = 3) experienced transient adverse effects ▪ 2 pts with worsening facial pain that fully resolved with short course steroids ▪ 1 pt with seizures due to cerebral edema that resolved with short-term use of antiepileptic medications ○ 5.9% (*n* = 3) developed permanent adverse effects ▪ 2 pts required long-term antiepileptic medications for seizures ▪ 1 pt developed panhypopituitarism ○ No new cranial nerve deficits after proton SRS
Petit et al., 2007 [62] (Acromegaly—Pituitary Adenoma)	Complete response: 59% (*n* = 13) No visual complications, seizure, brain injury, or secondary tumors were noted on follow-up imaging 38% (*n* = 8) developed new pituitary deficits
Petit et al., 2008 [63] (ACTH-producing Pituitary Adenoma)	Cushing’s disease cohort ○ 52% (*n* = 17) with complete response at a median time of 14 mos (range: 5–49 mos) ▪ No complete responder developed recurrent Cushing’s disease ○ 36% (*n* = 12) achieved stable urine cortisol levels; however, required continued medical therapy and did not have tumor regrowth ○ 12% (*n* = 4) with persistently elevated urine cortisol had radiographic evidence of disease progression and underwent subsequent transsphenoidal surgery Nelson’s syndrome cohort ○ 100% (*n* = 5) achieved a complete response with no evidence of biochemical or radiographic progression at a median of 9 years (range: 8–11 years) No visual complications or clinical evidence of brain injury was observed ○ New temporal lobe enhancement seen in 2 pts, which was asymptomatic and both received prior RT ○ 52% (*n* = 17) developed new hypopituitarism after proton SRS requiring administration of hormones with a median time of onset of 27 mos (range: 9–60 mos)
Vernimmen et al., 2005 [67] (Cerebral AVM)	<14 cc Cohort ○ 18 patients were available for follow-up evaluations ○ Obliteration in 67% (*n* = 12) ○ Partial response: 17% (*n* = 3) ≥14 cc Cohort ○ 28 patients were available for follow-up evaluations ○ Obliteration in 43% (*n* = 12) ○ Partial response: 21% (*n* = 7) One patient developed seizures within 24 h of receiving the first fraction and, subsequently, all patients with supratentorial AVM were started on prophylactic antiepileptic medications Late complications ○ 23% (*n* = 15) developed transient late side effects ▪ 80% (*n* = 12) required prolonged steroid therapy ▪ 80% (*n* = 12) made a complete recovery and had no permanent side effects ○ 6% (*n* = 4) developed permanent late grade 3 or 4 side effects ▪ 2 pts developed epilepsy ▪ 2 pts developed permanent neurologic deficits
Weber et al., 2003 [68] (Vestibular Schwannoma)	Tumor Control ○ 2-year: 95.3% (95% CI: 90.9–99.9%); 5-year: 93.6% (95% CI: 88.3–99.3%) ○ 1 patient required salvage GKRS for local progression 32.5 mos after proton SRS Extratumoral hemorrhage within choroid plexus requiring craniotomy in anticoagulated patient 19.1 months after treatment 3 pts (3.6%) underwent salvage shunting for clinical hydrocephalus at a median time of 7.6 months after proton SRS ○ 1 pt required a subsequent resection ○ 2 pts treated to 17.1 CGE ○ 1 pt treated to 13.3 CGE Hearing Preservation ○ Of the 24% (*n* = 21) who had serviceable hearing at time of proton SRS, 33.3% (*n* = 7) retained functional hearing ability with a median F/U of 31.8 mos Facial Neuropathy ○ Transient: 5% (*n* = 4) at a median of 2 mos after treatment ○ Permanent: 8.9% (*n* = 7) (3 pts HB Grade 2, 3 pts HB Grade 3; 1 pt HB Grade 4) with median time of onset of 5.1 mos ○ 2- and 5-year facial nerve function preservation of 91.1% (95% CI: 85–97.6%) Trigeminal Neuropathy ○ Transient dysfunction in 5% (*n* = 4) at a median of 6.1 mos ○ New permanent dysfunction in 10.1% (*n* = 8) at a median of 4.9 mos: 2 and 6 pts with “significant” and “mild” permanent neuropathy, respectively ○ 3 pts with preexisting dysfunction experienced resolution ○ 2- and 5-year trigeminal preservation rate of 89.4% (95% CI: 82–96.7%)
Barker et al., 2003 [97] (Intracranial AVM)	Hemiparesis (*n* = 24), visual field deficits (*n* = 11), cognitive dysfunction (*n* = 8), ataxia (*n* = 4), speech deficits (*n* = 3), hemisensory deficits (*n* = 2), hearing loss (*n* = 1) ○ Several patients had more than 1 of the above deficits ○ Three patients had a global deterioration in neurologic function ○ Four patients had a disabling increase in headache severity Complications presented at 0.2–6.8 years after proton SRS Actuarial incidence of complications ○ 1-year post treatment: 1.9% ○ 2-years post treatment: 3.3% ○ 3-years post treatment: 3.7% ○ 5-years post treatment: 4.2% ○ 10-years post treatment: 4.5% Median dose in patients with complications: 17 Gy Median dose in patients without complications: 10 Gy Treatment dose and treatment volume were inversely correlated with incidence of complications on univariate (*p* < 0.001 and *p* = 0.04, respectively) and multivariate (*p* < 0.001 and *p* < 0.001, respectively) Older patients (over median age of 31) were more likely to have complications on univariate (*p* = 0.03) and multivariate (*p* = 0.02) analysis Patients with a history of prior hemorrhage were more likely to have complications on multivariate analysis (*p* = 0.003) Sex and thalamic/brainstem location were not statistically significant predictors of development of complications
Silander et al., 2002 [66] (Cerebral AVM)	AVM Obliteration: total: 7 pts, intermediate: 3 pts, minimal: 1 pt, none: 4 pts, enlarged: 3 pts 1 pt (8 cc) with edema and midline shift within 1 year of treatment developed headache and no other neurologic symptoms and complete resolution after prolonged steroids 4 pts (15 cc, 15 cc, 13 cc, 1 cc) developed slight edema 9 pts had epilepsy prior to RT: 7 pts went on to become seizure-free, 2 pts had no change in seizure activity 2 pts had temporary worsening of headache and tiredness 2 months following RT 5 pts had new but non-permanent neurologic symptoms, such as parasthesias, headaches, and emotional distress in the first months following treatment 1 pt with continued emotional distress 1 pt with permanent increase in headache No reported worsening in neurological functioning 14 pts judged their overall health improved or unchanged since completing RT
Seifert et al., 1994 [64] (Cerebral AVM)	Neurologic symptoms after proton SRS by symptom ○ Improvement: 44.4% (*n* = 28) ○ Unchanged: 27% (*n* = 17) ○ Worsened 28.6% (*n* = 18) ▪ Increased seizure frequency: 38.9% (*n* = 7) ▪ Hemorrhage of irradiated AVM: 27.8% (*n* = 5) (fatal in 2 pts) ▪ Progressive neurologic deficits: 11.1% (*n* = 2) ▪ Progressive neurologic deterioration due to radiation-induced leukoencephalopathy: 22.2% (*n* = 4) Neurologic symptoms and obliteration after proton SRS by AVM size ○ <3 cm (*n* = 17) ▪ Symptoms: improvement: 76.4% (*n* = 13), unchanged: 11.8% (*n* = 2), worsened: 11.8% (*n* = 2) ▪ Obliteration: complete: 58.8% (*n* = 10), unchanged: 41.2% (*n* = 7) ○ 3–6 cm (*n* = 37) ▪ Symptoms: improvement: 32.4% (*n* = 12), unchanged: 35.2% (*n* = 13), worsened: 32.4% (*n* = 12) ▪ Obliteration: unchanged: 100% (*n* = 37) ○ >6 cm (*n* = 9) ▪ Symptoms: improvement: 33.3% (*n* = 3), unchanged: 22.2% (*n* = 2), worsened: 44.5% (*n* = 4) ▪ Obliteration: unchanged: 100% (*n* = 9) Neurologic symptoms and obliteration after proton SRS by SM Grade ○ Grade I and II (*n* = 7) ▪ Symptoms: improvement: 85.7% (*n* = 6), unchanged: 14.3% (*n* = 1) ▪ Obliteration: complete 100% (*n* = 7) ○ Grade III (*n* = 24) ▪ Symptoms: improvement: 54.2% (*n* = 13), unchanged: 37.5% (*n* = 9), worsened: 8.3% (*n* = 2) ▪ Obliteration: complete: 12.5% (*n* = 3), unchanged: 87.5% (*n* = 21) ○ Grade IV (*n* = 25) ▪ Symptoms: improvement: 24% (*n* = 6), unchanged: 20% (*n* = 5), worsened: 56% (*n* = 14) ▪ Obliteration: unchanged: 100% (*n* = 25) ○ Grade V (*n* = 2) ▪ Symptoms: unchanged: 100% (*n* = 2) ▪ Obliteration: unchanged: 100% (*n* = 2) ○ Grade VI (*n* = 5) ▪ Symptoms: improvement: 60% (*n* = 3), worsened: 40% (*n* = 2) ▪ Obliteration: unchanged: 100% (*n* = 5)
Steinberg et al., 1990 [98] (Cerebral AVM)	Clinical outcomes ○ Excellent: 46% ○ Good: 34% ○ Poor: 14% ○ Death: 6% 27 pts in “excellent” or “good” condition prior to SRS remained stable or improved neurologically 2 pts initially in “poor” condition who received prior fractionated RT died at 9 and 14 mos after SRS 7 pts experienced neurologic deterioration due to recurrent AVM hemorrhage 4 pts clinically worsened 3–12 mos after SRS

Abbreviations: ACTH: adrenocorticotropin hormone; AVM: arteriovenous malformation; CI: confidence interval; F/U: follow-up; GKRS: Gamma Knife radiosurgery; HB: House–Brackmann; HR: hazard ratio; mos: months; *n*: number of patients; pts: patients; RN: radiation necrosis; RT: radiation therapy; SM: Spetzler–Martin; SRS: stereotactic radiosurgery.

## Abbreviations

MDPI	Multidisciplinary Digital Publishing Institute
DOAJ	Directory of Open Access Journals
SRS	Stereotactic radiosurgery
FSRT	Fractionated stereotactic radiation therapy
WBRT	Whole brain radiation therapy
CNS	Central nervous system
STAR	Stereotactic alignment in radiosurgery
SOBP	Spread out Bragg Peak
IMPT	Intensity modulated proton therapy
DNA	Deoxyribonucleic acid
RBE	Relative biological effectiveness
Gy	Gray
MRI	Magnetic resonance imaging
CI	Confidence interval
USA	United States of America
cc	Cubic centimeters
HR	Hazard ratio
NSCLC	Non-small cell lung cancer
RCC	Renal cell carcinoma
HN	Head and neck
SCLC	Small cell lung cancer
GI	Gastrointestinal
GYN	Gynecologic
RT	Radiation therapy
KPS	Karnofsky Performance Status
BrM	Brain metastases
F/U	Follow up
STR	Subtotal resection
GTR	Gross total resection
ACTH	Adrenocorticotropic hormone
AVM	Arteriovenous malformation
CGE	Cobalt gray equivalent
SM	Spetzler–Martin
GR	Gardner–Robertson
HB	House–Brackmann
Pt	Patient
GKRS	Gamma Knife radiosurgery
NF2	Neurofibromatosis type 2
WHO	World Health Organization
